# The usefulness of a mathematical model of exposure for environmental risk assessment

**DOI:** 10.1098/rspb.2010.2667

**Published:** 2011-01-05

**Authors:** J. N. Perry, Y. Devos, S. Arpaia, D. Bartsch, A. Gathmann, R. S. Hails, J. Kiss, K. Lheureux, B. Manachini, S. Mestdagh, G. Neemann, F. Ortego, J. Schiemann, J. B. Sweet

**Affiliations:** 1 Oaklands Barn, Lug's Lane, Broome, Norfolk NR35 2HT, UK; 2 European Food Safety Authority (EFSA), GMO Unit, Largo Natale Palli 5/A, 43121 Parma, Italy; 3 National Agency for New Technologies, Energy and Sustainable Economic Development, Research Centre Trisaia, 75026 Rotondella, Italy; 4 Bundesamt für Verbraucherschutz und Lebensmittelsicherheit (BVL), Federal Office of Consumer Protection and Food Safety, Mauerstrasse 39-42, 10117 Berlin, Germany; 5 Centre for Ecology and Hydrology, Maclean Building, Benson Lane, Wallingford OX10 8BB, UK; 6 Plant Protection Institute, Szent István University, Pater K. 1, 2100 Gödöllő, Hungary; 7 Animal Biology Department, University of Palermo, Via Archirafi, 18, 90123 Palermo, Italy; 8 Büro für Landschaftsökologie und Umweltstudien, Muehlenweg 60, 29358 Eicklingen, Germany; 9 Centro de Investigaciones Biológicas (CSIC), Departamento de Biología de Plantas, Laboratorio Interacción Planta-Insecto, C/ Ramiro de Maeztu 9, 28040 Madrid, Spain; 10 Julius Kühn Institute, Federal Research Centre for Cultivated Plants (JKI), Institute for Biosafety of Genetically Modified Plants, Erwin-Baur-Strasse 27, 06484 Quedlinburg, Germany; 11 Sweet Environmental Consultants, 6 The Green, Willingham, Cambridge CB24 5JA, UK

We respond to the Comment of Lang *et al*. [1] regarding our mathematical model [2] of exposure of non-target Lepidoptera to *Bt*-maize pollen expressing Cry1Ab within Europe. Lang *et al*. remark on the degree to which the model was subject to uncertainty. Perry *et al*. [2] did indeed emphasize precaution: they made four separate decisions to model worst-case scenarios; identified six distinct sources of variability to which their results might be sensitive; and emphasized six different bases for the uncertainty of predictions. Lang *et al*. rightly emphasize the importance of identifying to which parameters the results of a model are most sensitive; Perry *et al*. should perhaps have emphasized more that the parameter to which their estimates of mortality were most sensitive was undoubtedly the variable measuring the rate of change of mortality with concentration/dose of the Cry1Ab protein (i.e. the slope of the probit/logit regression line; see below).

**Figure 1.**
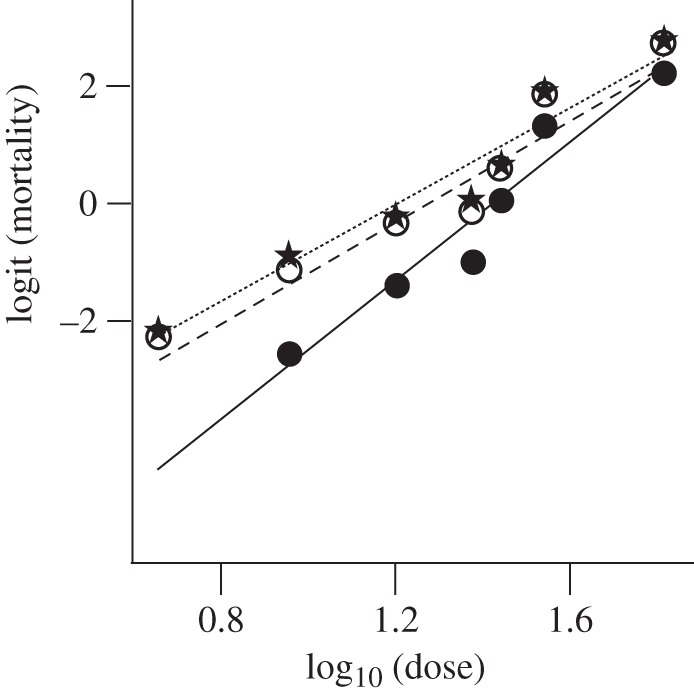
Logit-transformed observed percentage mortality from fig. 1 of [7] plotted against logarithmically transformed (base 10) dose (*Bt*-maize pollen consumed) from table 1 of [7], with fitted linear regression for 2 days (filled circles, solid line), 7 days (open circles, dashed line) and 14 days (stars, dotted line). All three regressions are highly significant (*p* < 0.001) with no indication of nonlinearity. The addition of a nonlinear term for curvature to the linear regressions was not significant for any of the three periods (*F*_1,3_ = 0.20, *F*_1,4_ = 1.83, *F*_1,4_ = 1.20, respectively).

Regarding the relationship between the toxicity of MON810 and *Bt*176 pollen, Lang *et al*. imply that the relationship between mortality and dose may be nonlinear. Regressions from bioassay should always be checked for nonlinearity, but there was no evidence of this in any of the extensive number of regressions of Saeglitz *et al*. [3,4] upon which our slope estimate was based. Of course, the standard transformation of mortality to probits (or logits) and the logarithmic transformation of concentration [5,6] are designed to achieve a linear regression; both papers cited by Lang *et al*. use this method. Data from one of these papers [7] were tested for nonlinearity; none was found, and no disproportionally higher mortality at low Cry1Ab concentrations could be verified (figure 1).

**Figure 2.**
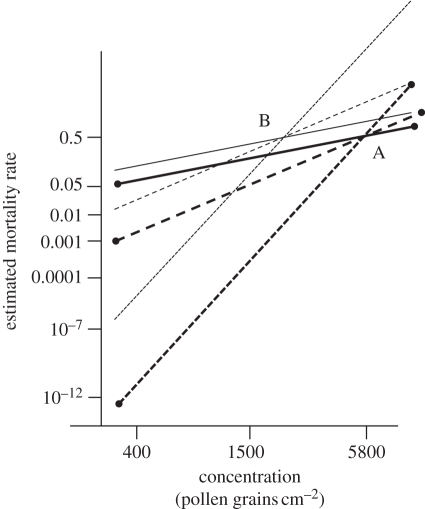
The probit regression line (thick solid line with button ends) for the LC50 of larvae of *Inachis io* assumed by Perry *et al*. [2] according to the slope value 1.095 estimated by Saeglitz *et al*. [3,4]. The assumed concentration of the Cry1Ab protein in pollen of maize MON810 is 31.05-fold less than that in maize *Bt*176. Shown for comparison are the lines expected if instead the different slope values of 2.25 (average of estimates of Farinós *et al*. [18]; thick long-dashed line with button ends) or 5.79 (estimated by Felke *et al*. [11]; thick short-dashed line with button ends) had been assumed. All three lines go through point A, the assumed LC50 of 5800 maize MON810 pollen grains cm^−2^, for which the mortality rate is 0.5. Within the crop, typical pollen concentrations are 10-fold less than this, and at the crop edge 30-fold less, and less still at distances into the margin, further from the edge. Also shown for comparison are three corresponding lines (thinner lines without button ends) with the same slope but different intercepts to the first three, representing the lines expected if the assumed concentration of the Cry1Ab protein in pollen of maize MON810 was only 12.2-fold less than that in maize *Bt*176. All three lines of this second set go through point B, for which the mortality rate is 0.5 and the assumed LC50 of 2283 grains cm^−2^. Estimated mortality is more sensitive to the differences in slopes than to the difference in intercepts.

Lang *et al*. are correct that Perry *et al*. used the range of published [8,9] values (12.2–78.9) to derive an average of the ratio of the concentration of the Cry1Ab protein expressed in pollen of maize *Bt*176 relative to that in maize MON810 for which a 31.05-fold difference was assumed. This is already likely to be a worst-case underestimate because, as Sears *et al*. [8] noted, the value of expression for MON810 was near the current level of detection by immunoassay. We do not agree with Lang *et al*.'s interpretation of the data from Nguyen [10]: they compared the smallest *Bt*176 value from 2002 to the largest MON810 value from 2003. The within-year ratio of *Bt*176 : MON810 was 64.8 in 2002 and 30.5 in 2003. The latter value, very close to that adopted by Perry *et al*., leads to larger mortality estimates for MON810 than the former, a further example of Perry *et al*.'s use of worst-case scenarios. Figure 2 shows the importance of the distinction between the intercept and slope of the probit/logit line in this issue. The conclusions of the model for risk management depend on the degree of estimated mortality. The conclusions are clearly highly sensitive to assumptions concerning the slope. They are sensitive, but much less so, to the intercept; it is the intercept that is governed by the *Bt*176 : MON810 ratio. Figure 2 demonstrates the effect of choosing an alternative worst-case value of the ratio (12.2) at the end of the range.

Regarding the assumption of equal susceptibility for the butterflies *Inachis io* and *Vanessa atalanta*, we apologize for the incorrect citation given by Perry *et al*. We acknowledge that data to compare the sensitivities of *I. io* and *V. atalanta* are very limited. We are aware of no evidence that the sensitivities differ; unpublished data from a single field experiment appear to suggest that they may not. Both Perry *et al*. and Lang *et al*. remarked on the need for further data on European Lepidoptera of conservation concern.

Lang *et al*. argue that the experimental methodology of Felke *et al*. [11] was likely to give results that underestimated true mortality. However, in many experiments with MON810, larvae have been exposed to longer time periods than those of Felke *et al*. [11]: 10 days [12]; 14–22 days [13]; 7 days [14]; 10–14 days [15]. These and other experiments with MON810 pollen have shown no negative effects when lepidopteran larvae were exposed to MON810 pollen alone. Furthermore, susceptibility to *Bt* toxin declines with age in older instars (e.g. [16]), so any potential for negative impacts of *Bt* pollen is reduced as the larvae develop. Lang *et al.* claim that fig. 1 of [7] demonstrates long-term effects (longer than 7 days) following a short acute dose of *Bt*. However, comparison of treatment and control in that figure appears to contradict rather than support their claim. In the period between 7 and 27 days, the mortality for a dose of 2.5 mg of *Bt* is only marginally (approx. 2%) greater than that of the control, while the mortalities for all other doses (1, 5, 7.5, 10, 20, 30 mg) are at least 5 per cent less than that of the control.

We fully agree with Lang *et al*. that sublethal effects should encompass fecundity and other parameters. However, many studies neglect to test parameters other than larval or pupal weight (e.g. [7]). Perry *et al*. emphasized that ‘our methods are subject to considerable uncertainty’, a caveat repeated in the final sentence of the Discussion.

Lang *et al*. stated that ‘all publications cited in Perry *et al*. in support of a possible reduction in exposure through behaviour of the larvae refer to *Danaus plexippus*’, but have perhaps overlooked Perry *et al*.'s text: ‘Both species [*V. atalanta* and *I. io*] are somewhat protected under field conditions from pollen deposition; the former species creates “leaf bags”, the latter builds webs (e.g. [17])’. We agree that the extent to which exposure is reduced through such behaviour is variable but it is surely not contentious to state that there is evidence for this for neonate larvae within Europe (see e.g. http://tristram.squarespace.com/home/2009/6/9/peacock-butterfly-caterpillars.html).

Regarding sensitivity analysis, it is important to allow for the fact that depositions of pollen in the field occur at far lesser concentrations than the LC50s for the three species considered by Perry *et al*. In consequence, as shown in figure 2, differing assumptions for the slope of the assumed probit (or logit) line will have little effect on the results for concentrations close to the LC50, but result in very large differences at concentrations around those expected within the crop or in the margin. Within the crop, the estimated mortality using the slope estimated by Felke *et al*. [11] is vanishingly small and such a value would be impossible to measure in field conditions. Even a doubling of the Saeglitz *et al*. [3,4] slope of close to 1.1 to the moderately small average 2.25 estimated by Farinós *et al*. [18] would result in roughly 10-fold decrease in estimated mortality. It is for these reasons that we consider that the consequence of this sensitivity completely outweighs any of the several effects claimed by Lang *et al*. to engender uncertainty and affect mortality estimates. We regard them as minor compared with the ‘safety margin’ factor of 8 **×** 10^−7^ by which Perry *et al*. inflated the estimated mortality through deliberate choice of a small value of the logit slope, designed to give worst-case mortality.

The value of the Perry *et al*. model is that it provides a transparent, structured and simple approach to exposure analysis that may be followed for other species and taxa in other settings, if sufficient data become available. Further, in its derivation of an integrated mortality–distance relationship, it offers the opportunity for relatively accurate laboratory-based estimation of mortality–dose relationships to supplement relatively inaccurate determinations of mortality in the field. We agree with Lang *et al*. that species' sensitivity to particular GM events that express different forms of Cry1 proteins is an important determinant of mortality; also that further data would be welcome on the mortality–dose relationships (particularly regarding the slopes) for a range of species, especially those of conservation concern. However, we disagree that we have been incautious regarding the implications of our results for conclusions regarding regulatory policy. We therefore reaffirm the robustness of our conclusion from our model that, after accounting for large-scale exposure effects, the ‘estimated environmental impact of MON810 pollen on non-target Lepidoptera is low’.
